# Angiosarcoma of the Scalp Presenting in Association With Borderline Malignant Phyllodes Tumour of the Breast

**DOI:** 10.1080/13577140310001644823

**Published:** 2003

**Authors:** Susan V. Harden, Richard Y. Ball, Adrian N. Harnett

**Affiliations:** 1 Department of Oncology Norfolk and Norwich University Hospital Colney Lane Norwich NR4 7UY UK; 2 Department of Histopathology Norfolk and Norwich University Hospital Norwich UK

## Abstract

Phyllodes tumours and angiosarcoma are both rare mesenchymal tumours. There are no reports of their coexistence in the literature except in families with germline p53 mutations. Here we report a case of an elderly woman who developed an extensive angiosarcoma of the scalp nearly 4 years after surgical removal of a borderline malignant phyllodes tumour of the breast. The scalp lesion was initially thought more likely to be a metastasis of her first rare tumour than a second equally rare primary tumour, but histologically this was not the case. The case and the literature are discussed.


*Sarcoma*, September/December 2003, *VOL. 7, NO. 3/4, 173–176*

Taylor & Francis healthsciences

CASE REPORT

### Angiosarcoma of the scalp presenting in association with borderline malignant phyllodes tumour of the breast

SUSAN V. HARDEN[1], RICHARD Y. BALL[2] & ADRIAN N. HARNETT[1]

*Departments of [1]Oncology and [2]Histopathology, Norfolk and Norwich University Hospital, Norwich, UK*

**Abstract**
Phyllodes tumours and angiosarcoma are both rare mesenchymal tumours. There are no reports of their coexistence in the literature except in families with germline p53 mutations. Here we report a case of an elderly woman who developed an extensive angiosarcoma of the scalp nearly 4 years after surgical removal of a borderline malignant phyllodes tumour of the breast. The scalp lesion was initially thought more likely to be a metastasis of her first rare tumour than a second equally rare primary tumour, but histologically this was not the case. The case and the literature are discussed.

**Key words:** *angiosarcoma, phyllodes tumour*

### Case report

An 81-year-old woman, who had previously been well, presented with a left breast mass. It had increased in size over several months but mammography suggested that it was benign. A $16 \times 15 \times 8.5$-cm phyllodes tumour was excised (Fig. 1). It showed variable cellularity. There was focal degeneration and calcification. No heterologous elements were recognised. While there was little cytological pleomorphism, mitoses were readily found (up to $44\,\text{mm}^{-2}$) and the neoplasm extended to the resection margin. The tumour was regarded as being of borderline type with a small risk of recurrence or metastasis. Subsequent studies showed that many of the stromal cell nuclei, particularly in the more cellular areas, showed moderate to strong immunostaining for p53 protein. Interestingly, similar immunoreactivity was present in the epithelial cells.

Four years later, she presented with a non-healing scalp laceration, with a history of having hit her head 2 months previously. However, the laceration was found to lie within an extensive bleeding nodular lesion, which extended over her scalp and onto her face (Fig. 2). A scalp haematoma was evacuated but as the lesion continued to bleed; debridement and skin grafting were performed by the plastic surgeons. The graft took well, but bleeding continued around the edges despite her having stopped anticoagulants (for atrial fibrillation). A biopsy of the lesion showed cutaneous angiosarcoma (Fig. 3). Its cells strongly immunostained for CD31 and CD34. Many of their nuclei showed moderate to strong immunoreactivity for p53 protein, as did some of the basal and parabasal keratinocytes of the epidermis and hair follicles. A CT scan showed no evidence of bony infiltration. She continued to deteriorate, with bleeding and disease progression, including bilateral cervical lymphadenopathy. Palliative radiotherapy to the scalp reduced the bleeding and discharge but she became anaemic, developed hyperkalaemia and died several months after presentation with the scalp lesion. Post mortem examination was not undertaken.

### Discussion

Phyllodes tumours are rare, constituting less than 1% of breast neoplasms.[1] Such tumours contain both stromal and epithelial components, of which the stromal elements are usually monoclonal and considered neoplastic.[2] Phyllodes tumours form a spectrum from benign (35–65%), through borderline (low-grade malignancy; 11–40%), to frankly malignant (18–35%) variants based on infiltrative margins, mitotic activity, stromal overgrowth and cellular atypia.[3–6] Considerable variation exists in the proportions of these subtypes in published series,

Correspondence to: A.N. Harnett, Department of Oncology, Norfolk and Norwich University Hospital, Colney Lane, Norwich NR4 7UY, UK. Tel.: +44-1603-289034; Fax: +44-1603-287463; E-mail: adrian.harnett@nnuh.nhs.uk

1357-714X print/1369–1643 online © 2003 Taylor & Francis Ltd
DOI: 10.1080/13577140310001644823

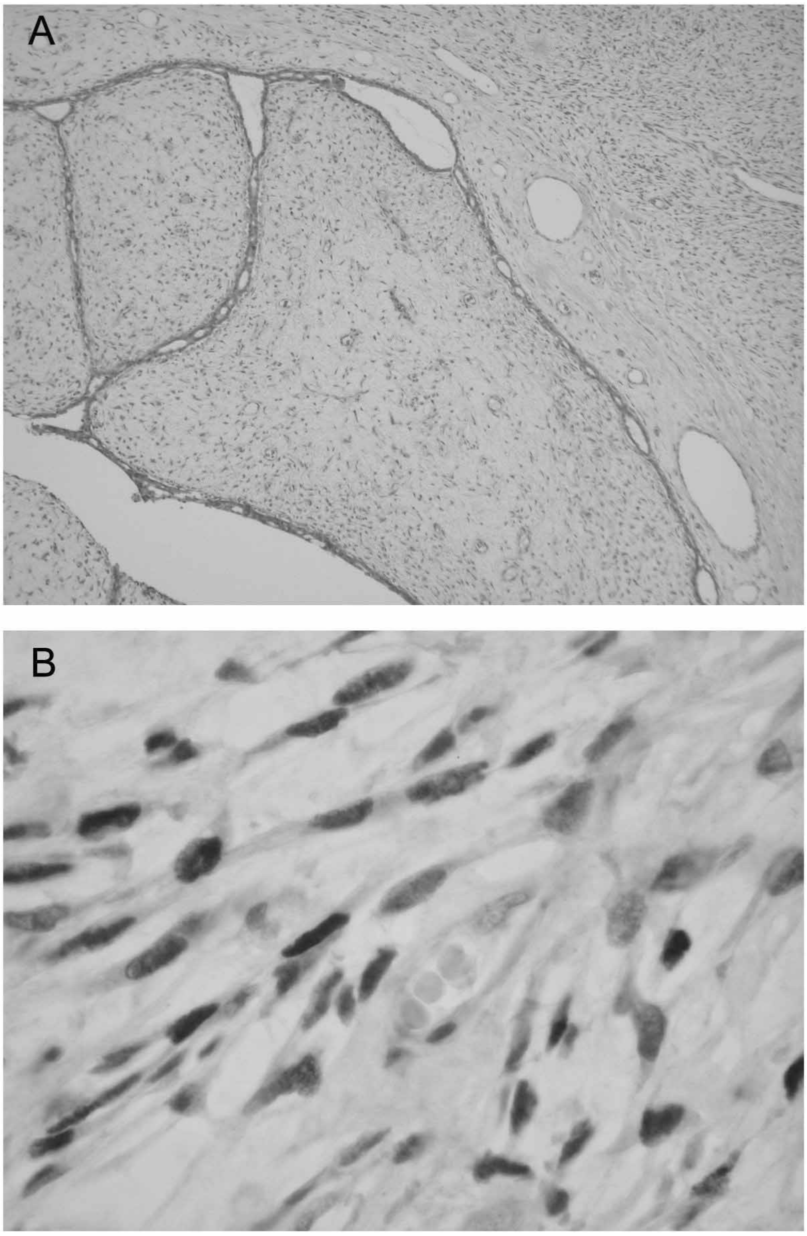

**Fig. 1.** *The borderline phyllodes tumour of the breast. (A) H&E-stained section; (B) p53-immunostained section showing strong nuclear staining of the stromal cells.*

probably because of a lack of standard histopathological interpretation.[5,6] Whilst tumour behaviour is often unpredictable, those tumours with more aggressive-looking histological appearances (i.e., borderline or frankly malignant) tend to recur locally and to metastasise.[1,7] Local recurrence is also associated with adequacy of surgical resection.[8] Local excision with adequate surgical margins is considered the standard treatment and routine axillary lymph node dissection is unnecessary[3] as lymph nodes are rarely involved. Adjuvant radiotherapy and chemotherapy are not usually necessary. However, postoperative radiotherapy is usually recommended for malignant tumours, treating as for a sarcoma. Metastasising potential has been associated with repeated local recurrence, increased mitoses and stromal overgrowth. The most common site for metastasis is the lungs, with sites such as bone, brain, pleura, soft tissue and skin occurring less frequently.[3,5,9,10]

There are several theoretical explanations for the development of the two neoplasms in this case, including: metastasis of heterologous elements in the phyllodes tumour; or development of two independent primary neoplasms as a result of inherited genetic abnormality; or acquired constitutional abnormality; or coincidence.

Metastatic spread to the scalp was considered likely clinically in this patient in view of her very large tumour, its focally positive surgical margins, relatively high mitotic rate and stromal overgrowth. However, histopathological examination did not

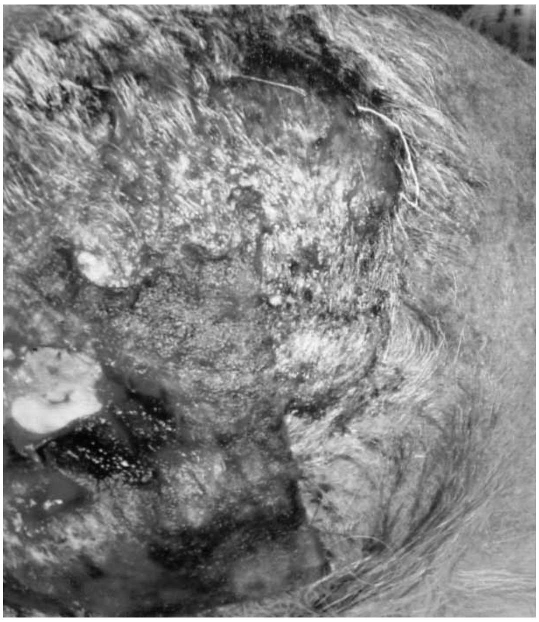

**Fig. 2.** *Extensive cutaneous angiosarcoma extending over the scalp with central laceration.*

support this, showing that the scalp lesion was a primary cutaneous angiosarcoma. Thorough examination of the breast tumour showed no evidence of heterologous elements. Angiosarcomatous foci can be seen in malignant phyllodes tumours, but were not present in this case. There are also no reports in the literature of phyllodes tumour metastases undergoing angiosarcomatous change, although heterologous stromal elements (bone, fat, cartilage) in the primary tumour can rarely become more prominent in metastases.[1,5,10]

Apart from young adults with germline p53 mutations, it is extremely unusual to see the development of two rare primary mesenchymal tumours in the same patient.[11,12] We are not aware of any reports of an association between phyllodes tumour and angiosarcoma outside of such cases. Malignant phyllodes tumours of the breast have been shown to be very strongly associated with germline p53 mutations. However, it is unlikely that our patient had a germline p53 mutation in view of her

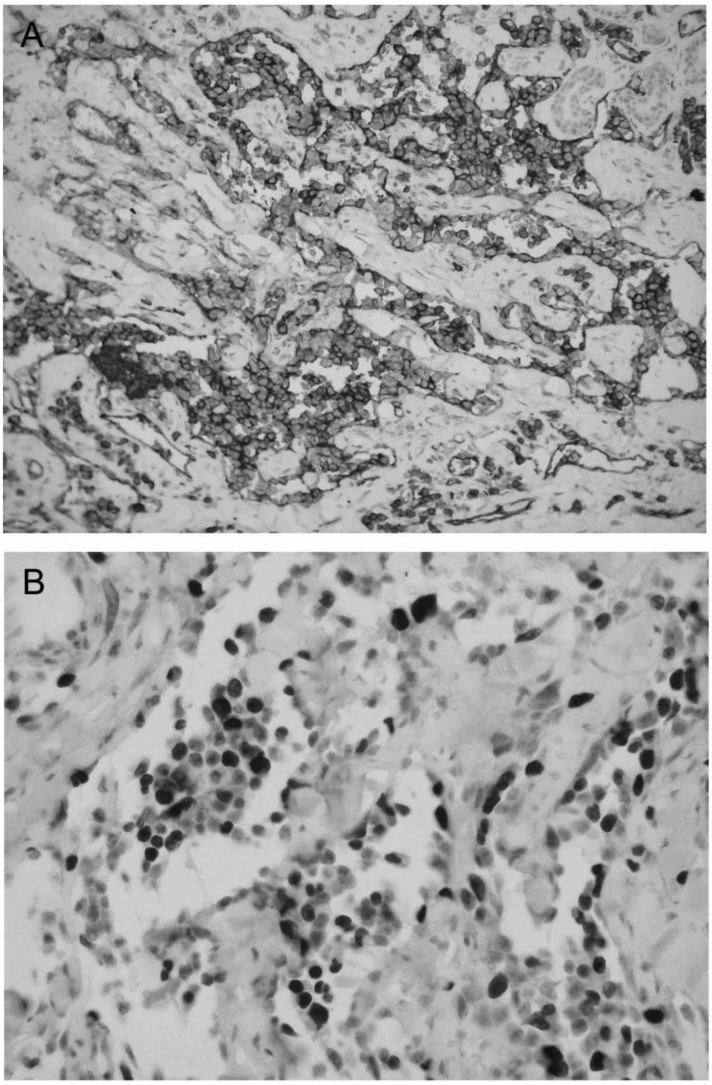

**Fig. 3.** *The cutaneous angiosarcoma. (A) CD31-immunostained section; (B) p53-immunostained section showing strong nuclear staining.*

advanced age at the time of diagnosis of both tumours and in the absence of previous history of malignancy. However, no analysis of her p53 genotype has been made, so this possibility, although remote, cannot be completely excluded. The immunostaining for p53 protein seen in the epithelial component of the phyllodes tumour and in keratinocytes is tantalising, but its significance is uncertain. It is possible that, with increasing age, other constitutional changes (such as immunological) took place, predisposing to the development of these two rare neoplasms. However, perhaps the most likely explanation is that there was no direct link between them.

The diagnosis of angiosarcoma of the scalp dramatically altered her prognosis. These scalp tumours tend to be highly aggressive, particularly when they are as extensive as in this case, often metastasising to adjacent lymph nodes. Both surgery and radiotherapy have low rates of local control because of the diffusely infiltrative nature of the condition and its potential for rapid multifocal and metastatic spread.[13,14] The best results for both are seen with small-volume, localised disease. Liposomal anthracyclines can cause tumour regression, but this elderly patient was too frail for this to be considered.

Whilst, in general, metastasis is more common than the development of a second rare primary neoplasm, this case report illustrates the fact that there are exceptions to the rule and emphasises the importance of undertaking a biopsy of suspected secondary lesions.

### References

1. Salvadori B, Cusumano F, Del Bo R, *et al.* Surgical treatment of phyllodes tumors of the breast. *Cancer* 1989; 63: 2532–6.
2. Noguchi S, Motomura K, Inaji H, Imaoka S, Koyama H. Clonal analysis of fibroadenoma and phyllodes tumor of the breast. *Cancer Res* 1993; 53: 4071–4.
3. Chaney AW, Pollack A, McNeese MD, *et al.* Primary treatment of cystosarcoma phyllodes of the breast. *Cancer* 2000; 89: 1502–11.
4. Christensen L, Schiodt T, Blichert-Toft M. Sarcomatoid tumours of the breast in Denmark from 1977 to 1987. A clinicopathological and immunohistochemical study of 100 cases. *Eur J Cancer* 1993; 13: 1824–31.
5. Reinfuss M, Mitus J, Duda K, Stelmach A, Rys J, Smolak K. The treatment and prognosis of patients with phyllodes tumor of the breast: an analysis of 170 cases. *Cancer* 1996; 77: 910–6.
6. Zissis C, Apostolikas N, Konstantinidou A, Griniatsos J, Vassilopoulos PP. The extent of surgery and prognosis of patients with phyllodes tumor of the breast. *Breast Cancer Res Treat* 1998; 48: 205–10.
7. Bissett D, Mallon E, Reed NS, George WD, Harnett AN. Cystosarcoma phylloides: heterogeneity in a rare tumour type. *J R Coll Surg Edinb* 1996; 41: 244–5.
8. Pietruszka M, Barnes L. Cystosarcoma phyllodes: a clinicopathologic analysis of 42 cases. *Cancer* 1978; 41: 1974–83.
9. Cohn-Cedermark G, Rutqvist LE, Rosendahl I, Silfversward C. Prognostic factors in cystosarcoma phyllodes. A clinicopathologic study of 77 patients. *Cancer* 1991; 68: 2017–22.
10. Hawkins RE, Schofield JB, Fisher C, Wiltshaw E, McKinna JA. The clinical and histologic criteria that predict metastases from cystosarcoma phyllodes. *Cancer* 1992; 69: 141–7.
11. Birch JM, Alston RD, McNally RJ, *et al.* Relative frequency and morphology of cancers in carriers of germline TP53 mutations. *Oncogene* 2001; 20: 4621–8.
12. Bot FJ, Sleddens HF, Dinjens WN. Molecular assessment of clonality leads to the identification of a new germ line TP53 mutation associated with malignant cystosarcoma phyllodes and soft tissue sarcoma. *Diagn Mol Pathol* 1998; 7: 295–301.
13. Sasaki R, Soejima T, Kishi K, *et al.* Angiosarcoma treated with radiotherapy: impact of tumor type and size on outcome. *Int J Radiat Oncol Biol Phys* 2002; 52: 1032–40.
14. Mark RJ, Tran LM, Sercarz J, Fu YS, Calcaterra TC, Juillard GF. Angiosarcoma of the head and neck. The UCLA experience 1955 through 1990. *Arch Otolaryngol Head Neck Surg* 1993; 119: 973–8.
